# Unraveling Hepatic Metabolomic Profiles and Morphological Outcomes in a Hybrid Model of NASH in Different Mouse Strains

**DOI:** 10.3390/antiox12020290

**Published:** 2023-01-27

**Authors:** Gabriel P. Bacil, Guilherme R. Romualdo, Priscila M. F. D. Piagge, Daniel R. Cardoso, Mathieu Vinken, Bruno Cogliati, Luís F. Barbisan

**Affiliations:** 1 Department of Pathology, Botucatu Medical School (FMB), São Paulo State University (UNESP), Botucatu 18618-689, Brazil; 2 Department of Structural and Functional Biology, Biosciences Institute, São Paulo State University (UNESP), Botucatu 18618-689, Brazil; 3 Department of Chemistry and Molecular Physics, São Carlos Institute of Chemistry (IQSC), University of São Paulo (USP), São Carlos 13566-590, Brazil; 4 Department of Pharmaceutical and Pharmacological Sciences, University of Vrije, 1090 Brussel, Belgium; 5 Department of Pathology, School of Veterinary Medicine and Animal Science, University of São Paulo (USP), São Paulo 05508-270, Brazil

**Keywords:** nonalcoholic fatty liver disease, nonalcoholic steatohepatitis, metabolomic profile, western diet, carbon tetrachloride

## Abstract

Nonalcoholic fatty liver disease (NAFLD) encompasses nonalcoholic steatohepatitis (NASH) and affects 25% of the global population. Although a plethora of experimental models for studying NASH have been proposed, still scarce findings regarding the hepatic metabolomic/molecular profile. In the present study, we sought to unravel the hepatic metabolomic profile of mice subjected to a hybrid model of NASH, by combining a Western diet and carbon tetrachloride administration, for 8 weeks, in male C57BL/6J and BALB/c mice. In both mouse strains, the main traits of NASH—metabolic (glucose intolerance profile), morphologic (extensive microvesicular steatosis and fibrosis, lobular inflammation, and adipose tissue-related inflammation/hypertrophy), and molecular (impaired Nrf2/NF-κB pathway dynamics and altered metabolomic profile)—were observed. The hepatic metabolomic profile revealed that the hybrid protocol impaired, in both strains, the abundance of branched chain-aromatic amino acids, carboxylic acids, and glycosyl compounds, that might be linked to the Nrf2 pathway activation. Moreover, we observed a strain-dependent hepatic metabolomic signature, in which the tricarboxylic acid metabolites and pyruvate metabolism were dissimilarly modulated in C57BL/6J and BALB/c mice. Thus, we provide evidence that the strain-dependent hepatic metabolomic profile might be linked to the distinct underlying mechanisms of NASH, also prospecting potential mechanistic insights into the corresponding disease.

## 1. Introduction

Non-alcoholic fatty liver disease (NAFLD) comprises a wide spectrum of chronic liver diseases, ranging from simple hepatic steatosis to non-alcoholic steatohepatitis (NASH), and affects almost 25% and 5% of the worldwide population, respectively [1]. NAFLD has become the leading cause of chronic liver disease and liver transplantations in the USA and is the fastest-growing cause of hepatocellular carcinoma (12-fold increase) (2002–2016) [1,2,3]. A population-based study indicated that adherence to a Western diet (WD) increases the risk of developing NAFLD by 56%. On the other hand, a Mediterranean vegetable/fruit-enriched diet reduces the risk of developing NAFLD by 23% [4]. The adverse effects of the WD are attributable to their high amount of saturated FA (SFA) and simple carbohydrates, in particular fructose and glucose, leading to “civilization diseases”, including obesity and diabetes [5]. NAFLD-related annual medical care costs reach up to $103 and €35 billion in the USA and Europe, respectively, with an expected doubling of NAFLD-related deaths in industrialized countries in the period 2016–2030 [6,7,8]. However, there is still no effective therapy approved by the health authorities, which could be attributed, at least in part, to gaps in the mechanistic understanding of NAFLD, which impeded to development of targeted therapies.

In an attempt to unravel the hallmarks of human NAFLD, a plethora of animal models has been introduced over the past decades focusing on the liver-adipose tissue (AT) axis dynamics [9]. Hybrid animal models (diet/chemically-induced) might offer a promising strategy for studying human-relevant NASH, as they manifest morphological characteristics (macrovesicular/microvesicular steatosis, lobular inflammation, and hepatic fibrosis) and molecular traits (hepatic transcriptomic profile), in a shorter-period protocol (12 weeks) [10]. In particular, the combination of WD and carbon tetrachloride (CCl_4_) promotes NASH development by establishing a pro-inflammatory/necrogenic hepatic milieu and accelerating the transition from simple steatosis to steatofibrosis [10,11]. However, it is still unclear how a strain-related susceptibility/resistance might influence outcomes and underlying molecular mechanisms of NASH, especially in such hybrid animal models.

Hepatic metabolomic profiles might be prospecting tools offering mechanistic insight into the biological processes triggered during the natural history of NASH. Furthermore, such metabolic signatures can assist in diagnosing and monitoring liver disease progression [12]. Indeed, serum/plasma metabolomic analysis showed that impaired metabolism of amino acids, carbohydrates, and fatty acids (FA) may drive NASH pathogenesis [13,14]. Similarly, the hepatic metabolomic profile of WD-fed mice displayed a clear difference in the glucogenic amino acid molecules, which is consistent with NASH-related impairment [15]. The present study was set up to identify strain-specific morphologic and metabolomic outcomes in a hybrid animal model of NASH.

## 2. Materials and Methods

### 2.1. Experimental Design

Male C57BL/6J and BALB/c mice were obtained from the School of Veterinary Medicine and Animal Science of the University of São Paulo (FMVZ, USP, São Paulo, Brazil), kept in the Experimental Research Unit (UNIPEX) of Botucatu Medical School of São Paulo State University (FMB, UNESP, Botucatu, Brazil). The animal study protocol was approved by the Botucatu Medical School/UNESP Ethics Committee on Use of Animals (CEUA) approval (Protocol number 1343/2020). Both mouse strains were chosen according to their relevance on preclinical studies. C57BL/6J shows a great susceptibility to the deleterious effects of chronic WD feeding and developing metabolic disorders, whereas BALB/c strains shows a greater susceptibility to hepatic fibrosis occurrence [16,17].

At week 8 following birth, animals were fed a WD [chow enriched with saturated fat/sucrose (200 g/Kg or 37.5 and 17% of total calories; Pragsoluções Biociências, Brazil) and a high-sugar solution [HSS, 55/45% weight/weight (*w*/*w*) proportion of d–fructose/d–glucose (Dinâmica, Brazil) or 23.1 and 18.9 g/L diluted in filtered water], or a basal diet [normocaloric chow (Nuvilab CR-1, Nuvital, Brazil) and tap water] for drinking, for 8 weeks (Figure S1). The nutritional composition of the WD and basal chows are depicted in Table S1. CCl_4_ and WD + CCl_4_ groups received weekly-increased intraperitoneal (i.p.) injections of a 10% diluted oil solution of CCl_4_ [0.25–1.50 µL/g of body weight (b.w.), 3×/week] protocol, for 8 weeks, while control and WD groups received corn oil vehicle, as previously described [18]. Animals were fasted for 12 h and euthanized by exsanguination under ketamine/xylazine anesthesia (300/45 mg/kg/b.w. i.p.) after 8 weeks of the interventions (WD/CCl_4_). Euthanasia occurred 48 h after the last i.p. injection of CCl_4_. Blood was collected in heparinized syringes from cardiac puncture, centrifuged (1503× *g*, 15 min), and serum samples were collected and stored at −80 °C for further analysis. At the necropsy, the liver and epididymal, peritoneal, and mesenteric AT were removed, weighted, and then hepatic samples from the left lobe and AT were fixed in 10% buffered formalin or snap-frozen into liquid nitrogen for further storage at −80 °C. The animals were kept (3 animals/cage) in a room with continuous ventilation, relative humidity (45–65%), controlled temperature (20–24 °C), and light/dark cycle 12:12 h, with ad libitum offering of chow and water. Body weight and food/water consumption were recorded once a week during the experimental period. This study was performed under the ARRIVE guidelines and all animal experiments received human care according to the “Guide for the Care and Use of Laboratory Animals” [19,20]. 

### 2.2. Biochemical Analyses

#### 2.2.1. Glucose Tolerance Test

Four days before euthanasia, mice were fasted for 12 h and submitted to a glucose tolerance test (GTT). Mice were weighed and blood samples were collected from the caudal vein in order to measure the glucose level in an automatic glucometer (Accu-Chek, Roche, Germany). Animals received a single i.p. injection of a water-diluted d-glucose solution (2 g/Kg b.w.) (Dinâmica, Brazil). Glucose levels were measured at 30, 60, 90, and 120 min. after injection, and the area under the curve (AUC) was calculated [21].

#### 2.2.2. Serum Alanine Aminotransferase Levels Determination

Alanine aminotransferase (ALT) serum levels were determined with a conventional kinetic assay, following the instructions provided by the manufacturers (Bioclin-Quibasa, Belo Horizonte, Brazil). ALT level assessment was performed with an automated spectrophotometer.

### 2.3. Histological Analyses

#### 2.3.1. NAFLD Activity Score and Lipid/Collagen Content Assessment

Formalin-fixed liver sections (5 µm) stained with hematoxylin and eosin (H&E) and Sirius red were used for evaluating the NAFLD Activity Score (NAS), and hepatic collagen content, respectively, according to previously established criteria [22], in 10 randomly selected fields/animal (200× magnification). The NAS was calculated for each mouse as the sum of the scores of macrovesicular steatosis, microvesicular steatosis, and hepatocellular hypertrophy, scored as 0 (<5% of the microscopic field), 1 (5–33%), 2 (33–66%), or 3 (>66%). Lobular inflammation was also evaluated, and animals were classified as displaying normal (0, <0.5 inflammatory foci/field), slight (1, 0.5–1.0 foci), moderate (2, 1.0–2.0 foci), severe (3, >2.0 foci) inflammation. Therefore, the sum of these parameters in each group could potentially range from 0 to 12. As such, 10 µm thick liver sections were obtained from snap-frozen samples (−80°C) embedded in an Optimal Cutting Temperature medium Tissue-Tek (Sigma Aldrich, St. Louis, MO, USA), and stained with Oil Red (Sigma Aldrich, St. Louis, MO, USA). A total of 10 photomicrographs/animal of oil red/Sirius red-stained sections were analyzed using software Leica Qwin V3 software (Leica Microsystems, Wetzlar, Germany), thereby screening the area (%) of lipid and collagen deposits. The blinded histopathologic evaluation was performed by the two experienced pathologists Professor Luís Fernando Barbisan and Professor Bruno Cogliati.

#### 2.3.2. Adipocyte Morphometry and Mast Cell Count

Five µm AT sections were stained with H&E and toluidine blue (0.05%, pH 4.0) (Sigma Aldrich, St. Louis, MO, USA), and were used for evaluating the size of adipocytes and mast cell (MC) density (number of mast cell/mm*^2^*), respectively, as previously described [23]. Photomicrographs of 10 randomly selected fields were acquired (200× magnification) and analyzed by ImageJ software (NIH, AR, USA). For adipocyte analysis, the extension Adiposoft for Image J (NIH, AR, USA) was used.

#### 2.3.3. Immunohistochemistry

Semi-quantitative immunostaining of Ki67 (i.e., a marker for cellular proliferation), CD68 (i.e., a marker of macrophages), α-smooth muscle actin (α–SMA) (i.e., a marker of activated hepatic stellate cell, HSC), and cleaved caspase-3 (casp3) (i.e., a marker of apoptotic cells) was performed in liver sections. Epididymal AT sections were subjected to CD68 immunostaining. Deparaffinated 5 µm sections in silane-covered slides were processed in view of antigen retrieval in a Pascal Pressure Chamber (Dako Cytomation, Denmark). Subsequently, blocking of endogenous peroxidase with 10% hydrogen peroxide solution was performed (15 min), followed by treatment with skim milk (60 min) and overnight incubation in a humidified chamber (4 °C) with primary antibodies directed against Ki67 (MA5-14520, 1:200 dilution, ThermoFisher, USA), CD68 (ab125212, 1:1000 dilution, Abcam, UK), α–SMA (ab124964, 1:500 dilution, Abcam, UK), and casp3 (5A1E, 1:50, dilution, Cell Signaling, Danvers, MA, USA) diluted in 1% bovine serum albumin solution. Following 3 wash steps with PBS (5 min. each), slides were incubated with single-step horseradish peroxidase (HRP)-polymer (EasyPath-Erviegas, Indaiatuba, Brazil) (20 min.). Reactions were visualized with 3′3-diaminobenzidine (DAB) chromogen (Sigma Aldrich, St. Louis, MO, USA), and counterstained with Harris hematoxylin. Photomicrographs of 10 randomly selected fields/animals (200× magnification) from immunostained liver sections for α–SMA were acquired and the relative area (%) assessment was performed with Leica Qwin V3 software (Leica Microsystems, Wetzlar, Germany). Similarly, CD68, Ki67, and casp3 analyses were performed by acquiring photomicrographs of 10 randomly selected fields/animals (200× magnification). The mean number of CD68-positive (CD68^+^) macrophages and Ki67-positive (Ki67^+^) and casp3-positive (casp3^+^) hepatocytes were expressed by area analyzed (mm*^2^*) using Image J software (NIH, Bethesda, MD, USA).

### 2.4. Molecular Analyses

#### 2.4.1. Protein Extraction and Immunoblotting

Liver samples from the left lobe (100 mg) were procured, stored at -80 °C, and homogenized in RIPA buffer (Cell Signaling, Danvers, MA, USA) with the addition of 1% of a protease inhibitor cocktail (Sigma Aldrich, St. Louis, MO, USA) followed by grinding with a Polytron mixer (Thermo Fisher Scientific, Waltham, MA, USA) (25,000 rpm, 10 s). Samples were subsequently stored at 4 °C for 2 h, and centrifuged (10,000× *g*, 4 °C, 30 min). Supernatants were collected for further protein quantification by the Bradford assay. For immunoblotting assays, 10 µL of the liver homogenate (total protein concentration of 7 µg/µL) was mixed with the same volume of Laemmli buffer, and heated (95 °C, 5 min). Next, samples were subjected to electrophoresis in 10.0% SDS-PAGE gel and transferred to a nitrocellulose membrane (Bio-Rad Laboratories, Hercules, CA, USA). Membranes were blocked with 5% skim milk solution diluted in Tris-tween solution for 1 h. Following 3 washing steps (5 min) membranes were incubated overnight with primary antibodies directed againstNrf2 (PA5-27282, 95–110 kDa, 1:1000, Thermo Fisher Scientific, Waltham, MA, USA), NF−κB (p65) (sc-372, 65 kDa, 1:1000, Santa Cruz Biotechnology, Dallas, TX, USA) and β-actin (sc-1615, 43 kDa, 1:1000, Santa Cruz Biotechnology, Dallas, TX, USA) diluted in TBS-T. After 5 washing steps, membranes were incubated with secondary antibodies for 2 h. Membranes were exposed to ECL Clarity Max (Bio-Rad Laboratories, Hercules, CA, USA) for immunodetection in a G:BOX Chemi System (Syngene, Cambridge, UK), with the software GeneSys (Syngene, Cambridge, UK). Semi-quantification was done in the ImageJ software (NIH, Bethesda, USA), with the protein of interest normalized against the housekeeping protein β-actin. Of note, the immunoblotting analysis was technically repeated 3x/each protein marker.

#### 2.4.2. Untargeted Metabolomics Analysis

For tissue homogenization and polar metabolites extraction, liver samples (200 mg) stored at −80 °C were placed into 2 mL RNase DNase free homogenization tubes containing ceramic beads with a diameter of 1.4 mm (matrix D, MP Biomedicals, CA, USA). Extraction solvent (chloroform/methanol/distilled water 1:2:1 *v*/*v*, according to the sample weight) was added to each tube and the tissues were then homogenized in a FastPrep-24 homogenizer (MP Biomedicals, CA, USA) for two cycles at 4 m/s (30 seg. Each). After homogenization, the samples were added 200 mL of chloroform for phase separation and centrifuged at 4 °C and 1500× *g* for 15 min, and supernatants, hydroalcoholic aqueous phase, were collected and concentrated in a SpeedVac Vacuum Concentrator (Thermo Fisher Scientific, Waltham, MA, USA) and stored at −80 °C until metabolomic analyses. NMR spectra were conducted at 298 K on a 500 MHz (11.7 T) Agilent DD2 spectrometer with a 5 mm OneNMR probe with gradient capability. The spectra were acquired for proton NMR using the PRESAT pulse sequence for the residual water signal suppression, 32 K data points, with a spectral width of 16 ppm, an acquisition time of 4.089 seg., were acquired a fixed receiver gain of 40, a recycle delay of 33 seg. (5*T1), dummy scans of 2, an accumulation of 128 transients. FIDs were multiplied by a 0.3 Hz exponential multiplication function prior to the Fourier transform. Phase and baseline corrections were carried out within the instrument software, and the reference standard (DSS-d6) signal was calibrated at δ 0.00 ppm. The 1D spectra were assigned using the Chenomx NMR Suite software as a database supported by literature and the 2D NMR spectra (gCOSY, gHSQC) were obtained for quality control samples. Metabolite peaks were integrated and quantified relative to DSS−D6 0.7 mM using Chenomx software for quantification. Following the NMR profiling, an enrichment analysis was performed with the MetaboAnalyst 5.0 platform and KEGG database (metabolites setup), to understand the molecular pathways associated with the hepatic metabolomic profile.

### 2.5. Statistical Analyses

GTT data were measured and analyzed by two-way ANOVA. Metabolomic profiles were analyzed by partial least squares − discriminant analysis (PLS−DA) followed by the VIP score determination (VIP ≥ 1.0). Other data were analyzed by one-way ANOVA or Kruskal-Wallis, followed by a post hoc Tukey’s test. Differences were considered significant when *p* < 0.05. Data were presented as mean ± standard deviation (S.D.) or median (maximum/minimum). For that, the software GraphPad Prism 6.01 (GraphPad, San Diego, CA, USA) was applied.

## 3. Results

### 3.1. General Findings

During the 8 weeks-hybrid protocol, the WD + CCl_4_ groups displayed reduced chow (*p* < 0.0001, for both mouse strains) and increased HSS intake (*p* < 0.0001, for both mouse strains) (Figure S2A,B,D,E), compared to control and CCl_4_ counterparts in both mouse strains, whereas no differences were observed in body weight evolution (Figure S2C,F). At necropsy, a yellowish and rough liver surface was observed in the WD + CCl_4_ groups of both strains (Figure S3). WD + CCl_4_ groups showed reduced final body weight compared to WD counterparts (*p* < 0.0001, for both mouse strains) (Figure S4A,F), which is consistent with the adverse effects of CCl_4_. However, the hybrid protocol increased both absolute (*p* < 0.0001) (Figure S4B,G) and relative liver weights (*p* < 0.0001) only in the BALB/c strain (Figure S4C,H). In agreement with the body weight findings, the WD groups showed increased absolute (*p* < 0.0001, for both mice strains) (Figure S4D,I) and relative total fat weight (*p* = 0.0002 and *p* < 0.0001, respectively) (Figure S4E,J), suggesting that the CCl_4_ protocol impairs fat weight gain of mice fed a WD protocol. We have previously showed that the ALT levels are more sensitive to the weekly-increased doses of a CCl_4_-induced models, rather than aspartate aminotransferase (AST) levels [18]. Thus, we assessed the serum levels of ALT as a biomarker for liver damage and as expected, the ALT levels were increased in the WD + CCl_4_ groups (*p* = 0.0020 and *p* = 0.0002, respectively) (Figure S5A,D), compared to the control counterparts. In addition, the mortality rate in CCl_4_ and WD+CCl_4_ groups reached around 17% and 6% in C57BL/6J, and 23% and 5% in BALB/c, respectively.

### 3.2. The Hybrid Model Induces a Strain-Dependent Glucose Intolerance 

In C57BL/6J mice, but not in BALB/c mice, the WD + CCl_4_ group featured increased glucose levels by 60 min. after glucose injection (*p* < 0.0001) (Figure S6A). Accordingly, the WD + CCl_4_ group displayed a higher AUC (*p* < 0.0001) (Figure S6B) compared to the control group. On the other hand, in BALB/c mice, only the WD group featured increased glucose levels, after 30 and 60 min. of glucose administration (*p* < 0.0001) (Figure S6C), compared to other groups, which was also reflected by the enhanced AUC (*p* = 0.0122) (Figure S6D). These findings suggest that the strain-dependent effects of the hybrid model might be related to the different sensibility of mouse strains.

### 3.3. The Hybrid Model Enhances NAS and Induces Lipid Deposition

**Figure 1:**
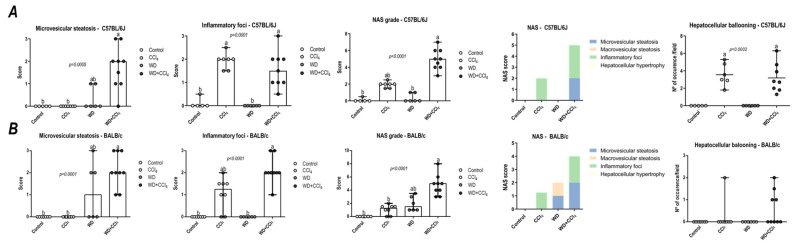
NAFLD activity score (NAS) of C57BL/6J and BALB/c mice subjected to the hybrid model of NASH. (**A**,**B**) Macrovesicular steatosis grade (0–3), microvesicular steatosis grade (0–3 grade), hepatocellular hypertrophy (0–3), inflammatory foci (0–3 grade), and final NAS grade (0–12 grade), parameters contribution for NAS grading, and hepatocellular ballooning occurrence of C57BL/6J and BALB/c, respectively. Data were analyzed by Kruskal-Wallis and Tukey post hoc test and presented as median (maximum and minimum). Different letters correspond to significant differences (*p* < 0.05) among groups. Control and WD: 5 or 7 animals/group; and CCl_4_ and WD + CCl_4_: 9 animals/group. WD: a high-fat/sucrose chow (20% of fat/sucrose) and high-sugar solution (HSS, D-fructose/D-glucose or 23.1 and 18.9 g/L) for drinking. CCl_4_: intraperitoneal (i.p.) injections of 10% diluted oil solution of CCl_4_ (0.25–1.50 µL/g of body weight, 3×/week).

**Figure 2:**
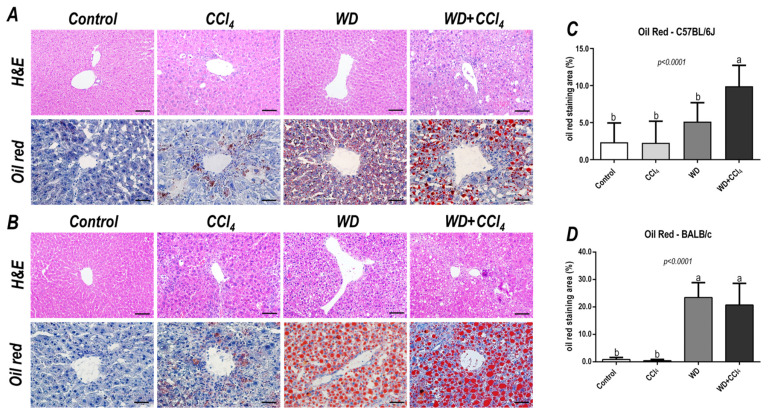
Representative photomicrographs (200× objective, scale bar = 50 μm) of hepatic sections stained with hematoxylin & eosin (HE) and oil red (400× objective, scale bar = 20 μm) of (**A**) C57BL/6J and (**B**) BALB/c subjected to the hybrid model of NASH. (**C**,**D**) Morphometric analysis of hepatic lipid content in C57BL/6J and BALB/c mice. Data were analyzed by one-way ANOVA and Tukey post hoc test. Data were presented as mean ± standard deviation (S.D.). Different letters correspond to significant differences (*p* < 0.05) among groups. Control, CCl_4_, WD, WD + CCl_4_: 5 animals/group. WD: a high-fat/sucrose chow (20% of fat/sucrose) and high-sugar solution (HSS, D-fructose/D-glucose or 23.1 and 18.9 g/L) for drinking. CCl_4_: intraperitoneal (i.p.) injections of 10% diluted oil solution of CCl_4_ (0.25–1.50 µL/g of body weight, 3×/week).

In both C57BL/6J and BALB/c mice, the hybrid model triggered a microvesicular steatosis profile (*p* = 0.0005 and *p* = 0.0001, respectively) (Figure 1) − 55% (5/9) and 80% (7/9) displayed high score steatosis, respectively—and pronounced inflammatory foci occurrence (*p* = 0.0001 and *p* < 0.0001, respectively) (Figure 1) − 45% (4/9) and 90% (8/9) presented a high score of inflammatory foci, respectively (Figure 2A,B). Hence, the WD + CCl_4_ C57BL/6J and BALB/c groups presented a higher final NAS compared to their counterparts (*p <* 0.0001, for both mouse strains) (Figure 1). The hepatocellular ballooning occurrence, however, was a C57BL/6J-specific characteristic and was frequently found in CCl_4_-receiving groups (*p* = 0.0002) (Figure 1). Additionally, only the WD + CCl_4_ groups manifested simultaneous occurrence of hepatic steatosis and lobular inflammation (Figure 2A,B), matching the signature traits of NASH (Supplementary Figure S7), rather than simple steatosis as observed in WD groups. In agreement with these findings, the Oil red analysis showed a strain-dependent response in the hybrid model of NASH. The WD + CCl_4_ groups displayed pronounced lipid abundance (*p* < 0.0001, for both mouse strains) (Figure 2), compared to the control counterparts, which is consistent with the steatosis grading (Figure 1). However, an additive effect of the WD and CCl_4_ protocols led to increased lipid accumulation only in the C57BL/6J strain (Figure 2C). On the other hand, in the BALB/c strain, similar hepatic lipid levels were found in WD-fed mice (Figure 2D). Taken together, both strains showed signature traits of high-grade NASH, although only in C57BL/6J mice the CCl_4_ protocol accelerated WD-related hepatic lipid accumulation.

### 3.4. The Hybrid Model Enhances the Hepatic Collagen Content, α−SMA Levels and CD68 Cells Density

**Figure 3:**
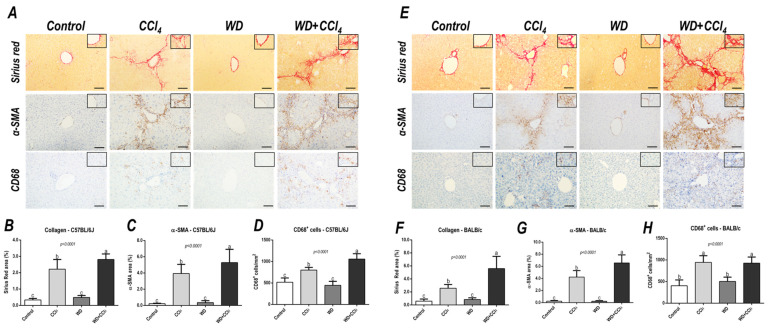
Representative photomicrographs of hepatic sections stained with Sirius red and immunoreacted for α-smooth muscle actin (α−SMA) and CD68 (200^×^ objective, scale bar = 50 μm) of (**A**) C57BL/6J and (**E**) BALB/c subjected to the hybrid model of NASH. (**B**,**F**) Assessment of the collagen content by Sirius red stain relative area (%). (**C**,**G**) Assessment of the α-SMA relative area (%) and (**D**,**H**) the number of CD68^+^ cells/mm^2^ (200^×^ objective, scale bar = 50 μm). Data were analyzed by one-way ANOVA and Tukey post hoc test. Data were presented as mean ± standard deviation (S.D.). Different letters correspond to significant differences (*p* < 0.05) among groups. Control and WD: 5–7 animals/group; and CCl_4_ and WD + CCl_4_: 9 animals/group. WD: a high-fat/sucrose chow (20% of fat/sucrose) and high-sugar solution (HSS, d−fructose/d−glucose or 23.1 and 18.9 g/L) for drinking. CCl_4_: intraperitoneal (i.p.) injections of 10% diluted oil solution of CCl_4_ (0.25–1.50 µL/g of body weight, 3×/week).

The hybrid model of NASH enhanced the hepatic collagen content (*p* < 0.0001, for both mouse strains) and HSC activation (*p* < 0.0001, for both mouse strains) (Figure 3) in both mouse strains, compared to other groups, suggesting that the combination of WD with weekly-increased doses of CCl_4_ accelerates fibrosis by enhancing HSC activity. Likewise, CD68^+^ cell density was enhanced in the WD + CCl_4_ group (*p* < 0.0001) (Figure 3A,C) of the C57BL/6J strain, compared to the other groups. In the BALB/c strain, the CCl_4_-receiving groups showed increased CD68^+^ cell density (*p* < 0.0001) (Figure 3B,D), compared to the control group. Thus, the CCl_4_ accelerates the WD-related hepatic injury, enhancing HSC activation and collagen deposition, whereas increasing CD68^+^ cell density, a well-known potential driver of NASH, was a C57BL/6J strain-specific event, indicating potential dissimilar mechanisms of developing NASH in mouse strains.

### 3.5. The Hybrid Model Enhances the Number of Ki67^+^ and casp3^+^ Hepatocytes 

**Figure 4:**
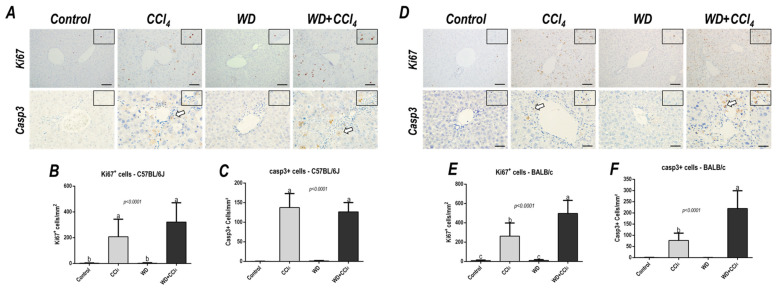
Representative photomicrographs of hepatic sections immunoreacted for Ki67 and casp3 (200× objective, scale bar = 50 μm) of (**A**) C57BL/6J and (**D**) BALB/c subjected to the hybrid model of NASH. (**B**,**E**) Assessment of the number of Ki67^+^ and (**C**,**F**) casp3^+^ cells/mm^2^ (200× objective, scale bar = 50 μm). Data were analyzed by one-way ANOVA and Tukey post hoc test and presented as mean ± standard deviation (S.D.). Different letters correspond to significant differences (*p* < 0.05) among groups. Control and WD: 5–7 animals/group; and CCl_4_ and WD + CCl_4_: 9 animals/group. WD: a high-fat/sucrose chow (20% of fat/sucrose) and high-sugar solution (HSS, d−fructose/d−glucose or 23.1 and 18.9 g/L) for drinking. CCl_4_: intraperitoneal (i.p.) injections of 10% diluted oil solution of CCl_4_ (0.25–1.50 µL/g of body weight, 3×/week).

Effects of the hybrid model of NASH on the Ki67^+^ and casp3^+^ hepatocyte density were assessed. In the C57BL/6J strain, the CCl_4_-treated groups showed an increased number of both Ki67^+^ (*p* < 0.0001) and casp3^+^ hepatocytes (*p* < 0.0001) (Figure 4A,C). On the other hand, only in the BALB/c strain, the WD + CCl_4_ group featured enhanced density of Ki67^+^ (*p* < 0.0001) and casp3^+^ (*p* < 0.0001) hepatocytes (Figure 4B,D), compared to the counterparts. These findings suggest that, in the BALB/c strain, a distinct response to the hybrid model of NASH might be linked to lipotoxicity-related underlying mechanisms in hepatocytes.

### 3.6. The Hybrid Model Impairs in a Strain-Dependent Manner the p65−NF−κB/Nrf2 Pathway

**Figure 5:**
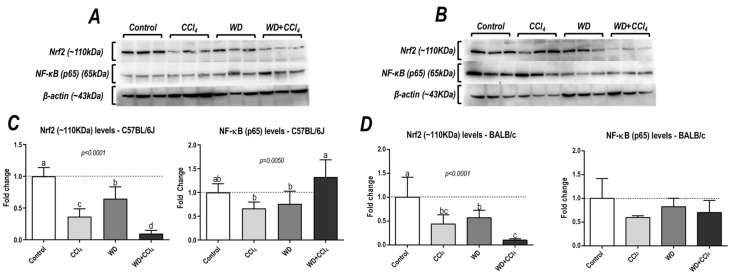
Analysis of hepatic protein levels of Nrf2 and p65 subunit of nuclear kappa B (p65−NF− κB) of (**A**) C57BL/6J and (**B**) BALB/c. (**C**,**D**) Semi-quantitative densitometric analysis of Nrf2 and p65−NF−κB levels in C57BL/6J and BALB/c, respectively. Data were analyzed by one-way ANOVA and Tukey post hoc test and presented as mean ± standard deviation (S.D.). Different letters correspond to significant differences (*p* < 0.05) among groups. Control, CCl_4_, WD, CCl_4_, and WD + CCl_4_: 6 animals/group WD: a high-fat/sucrose chow (20% of fat/sucrose) and high-sugar solution (HSS, d−fructose/d−glucose or 23.1 and 18.9 g/L) for drinking. CCl_4_: intraperitoneal (i.p.) injections of 10% diluted oil solution of CCl_4_ (0.25–1.50 µL/g of body weight, 3×/week).

The hybrid model of NASH caused a striking decrease in protein levels of Nrf2 in both C57BL/6J and BALB/c mouse strains (*p* < 0.0001, for both mouse strains) (Figure 5), a well-known modulator of the antioxidant cell line defense, which might be linked to NASH emergence. Surprisingly, only in the C57BL/6J strain, the WD + CCl_4_ group presented increased protein levels of p65−NF−κB (*p* = 0.0050) compared to other groups (36% compared to the control group) (Figure 5A,C), indicating that the hybrid model triggers a master regulator of the pro-inflammatory underlying mechanisms of NASH.

### 3.7. The Hybrid Model Modulates the Hepatic Metabolomic Profile

**Figure 6:**
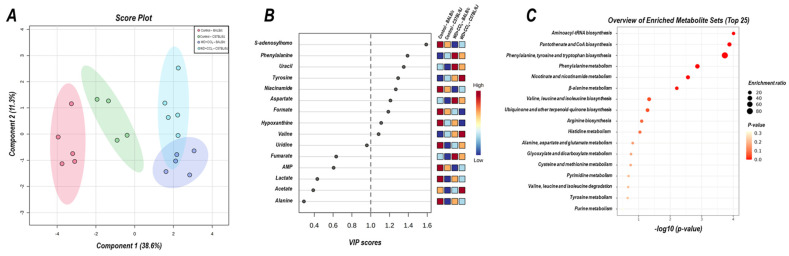
Metabolomic hepatic profile of C57BL/6J and BALB/c mice subjected to hybrid NASH model vs. controls. (**A**) 2D partial least squares-discriminant analysis (PLS−DA) demonstrating the clustering profile between hepatic samples of C57BL/6J and BALB/c mice (vs. control counterparts). (**B**) Differential abundance of metabolites (variable importance in projection, VIP ≥ 1.0). (**C**) Enrichment analysis of the altered abundant metabolites. Control: 5; WD + CCl_4_: 4–5 animals/group. WD: a high-fat/sucrose chow (20% of fat/sucrose) and high-sugar solution (HSS, d−fructose/d−glucose or 23.1 and 18.9 g/L) for drinking. CCl_4_: intraperitoneal (i.p.) injections of 10% diluted oil solution of CCl_4_ (0.25–1.50 µL/g of body weight, 3×/week).

**Figure 7:**
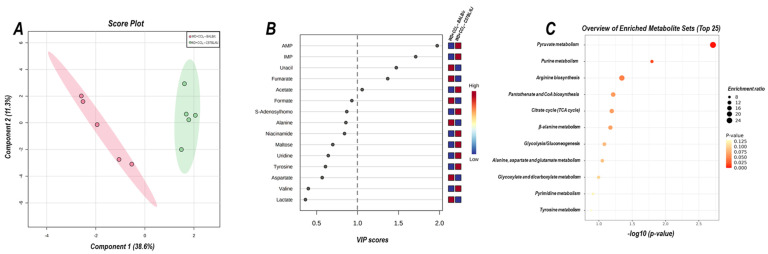
Strain differences (C57BL/6J vs. BALB/c) in the hepatic metabolomic profile of WD + CCl_4_ groups. (**A**) 2D partial least squares-discriminant analysis (PLS−DA) demonstrates the clustering profile between hepatic samples of C57BL/6J and BALB/c mice. (**B**) Differential abundance of metabolites (variable importance in projection, VIP ≥ 1.0). (**C**) Enrichment analysis of the altered abundant metabolites. WD + CCl_4_: 4–5 animals/group. WD: a high-fat/sucrose chow (20% of fat/sucrose) and high-sugar solution (HSS, d−fructose/d−glucose or 23.1 and 18.9 g/L) for drinking. CCl_4_: intraperitoneal (i.p.) injections of 10% diluted oil solution of CCl_4_ (0.25–1.50 µL/g of body weight, 3×/week).

The 2D PLS−DA analysis (WD + CCl_4_ vs. control, considering both mouse strains) revealed clear clustering among WD + CCl_4_ and control groups (Figure 6A), evidencing that the hybrid model of NASH modulates the hepatic metabolomic profile regardless of the mouse strain. Indeed, the WD + CCl_4_ groups displayed increased levels of phenylalanine, tyrosine, aspartate, and valine amino acids as well as reduced levels of S-adenosylhomocysteine (SAH), niacinamide, formate, and hypoxanthine (Figure 6B). Enrichment analysis revealed the chemical class annotations in the NASH model related to amino acids and peptides, pyridine carboxylic acids, and glycosyl compound dynamics. Furthermore, aminoacyl-tRNA biosynthesis, pantothenate and coenzyme A (CoA) biosynthesis, phenylalanine, tyrosine, and tryptophan biosynthesis, and phenylalanine metabolism were altered in the hybrid NASH model (Figure 6C), suggesting a role for these metabolites and metabolic pathways in NASH pathogenesis. In the next step, WD + CCl_4_ groups were compared in order to identify the strain-dependent potential underlying mechanism of NASH. As such, two signature hepatic metabolomic profiles were captured by the 2−D PLS−DA analysis (C57BL/6J × BALB/c) (Figure 7A). Thus, C57BL/6J mice presented a decreased abundance of fumarate and uracil, whereas BALB/c mice displayed lower levels of adenosine monophosphate (AMP), inosine 5′- monophosphate (IMP), and acetate (Figure 7B). The enrichment analysis suggested that these strain-dependent metabolomic profiles were related to the tricarboxylic acid cycle (TCAC) metabolites and that pyruvate metabolism and purine metabolism might underlie the dissimilar effects of the hybrid model of NASH in C57BL/6J and BALB/c mice (Figure 7C).

### 3.8. The Hybrid Model Modulates the Adipocyte Size and Increases at Inflammatory Cells Density

We also assessed adipocyte size and inflammatory cell density in the AT. Surprisingly, only in BALB/c strain, the hybrid protocol increased the adipocyte size (*p* < 0.0001) (Figure S8) and MC cells density (*p* = 0.0083) (Figure S8B,D), whereas the CD68^+^ cell density was increased in both WD + CCl_4_ groups (*p* = 0.0432 and *p* = 0.0004, respectively) (Figure S8) of C57BL/6J and BALB/c mice.

## 4. Discussion

The present study was set up to study strain-specific morphologic and metabolomic effects in a hybrid model of NASH in C57BL/6J and BALB/c. After 8 weeks of the hybrid protocol of NASH, several morphologic, metabolic, and molecular parameters showed a strain-dependent susceptibility, which is consistent with variations in metabolomic profiles. A yellowish, rough, and irregular liver surface was observed in WD-fed mice in both strains, suggesting the accumulation of lipid/collagen similar to previous findings of other murine models of NASH [10,24,25]. Metabolic disorders − especially obesity, glucose intolerance, and insulin resistance − are not frequently observed in chemically-induced models of NASH, yet is a major phenotypical aspect of NAFLD in patients [1,26]. Surprisingly, distinct effects of the combination of WD and CCl_4_ protocols targeting the AT differed in both strains. C57BL/6J mice displayed a glucose intolerance profile and CD68^+^ cell infiltration in the AT, whereas adipocyte hypertrophy and enhanced inflammatory cells − both CD68^+^ and MC − density were prominent in the BALB/c strain. During NASH development, an interplay between the liver and AT occurs, impacting glucose/insulin metabolism and systemic low-grade inflammation [27]. In WD-fed mice, AT macrophages (ATM) exert a pivotal modulation of the AT milieu by inducing the synthesis of TNF-α, IL-1β, and IL-6 and contributing to hepatic inflammatory cell infiltration, whereas depleting ATM alleviates NASH. Moreover, transplanting epididymal AT from obese mice into lean acceptor mice enhances hepatic inflammatory subset cells and contributes to low-grade inflammation and NASH progression [28].

The combination of WD and CCl_4_ mimicked a high-grade NASH in both mouse strains, resulting in high scores of hepatic microvesicular steatosis and lobular inflammation in addition to abundant levels of hepatic collagen/lipid deposition and HSC activation. However, a dissimilar morphologic profile was observed according to mouse-strain. Our findings indicate that both mice are susceptible to WD and CCl_4_-associated fibrosis and HSC activation, but the hepatic combination of WD and CCl_4_ enhanced the hepatic lipid content in C57BL/6J mice, only. Moreover, both CD68- and casp3-associated mechanisms might be linked to potential distinct mechanisms of NASH emergence in C57BL/6J and BALB/c, respectively. Under conditions of SFA overload, hepatocytes might suffer phenotypical changes that induce the synthesis of extracellular vesicles (EV) with pro-inflammatory content (e.g., IL-6 and IL-1β), triggering the activation of HSC and the recruitment of inflammatory cells. Furthermore, SFA exposure activates reticulum stress-related mechanisms due to the high content of lipotoxic molecules, like ceramides, thereby increasing levels of casp3, which burgeons into the onset of apoptosis [29,30,31,32]. Likewise, CCl_4_ is a hepatotoxin that undergoes biotransformation yielding trichloromethyl radical (CCl_3_), which causes lipid peroxidation, apoptosis, and compensatory proliferation of hepatocytes, by the cytochrome P450 activity, also triggering oxidative stress-related pathways and inducing inflammatory cells recruitment [33,34]. It has been previously reported that a low-dose CCl_4_ protocol accelerated NASH emergence in a WD-induced murine model, by exacerbating the hepatic collagen deposition and lipotoxic effect [35]. Indeed, the hepatic injuries caused by CCl_3_**.** increase the activation of HSC − drivers of type I and III collagen synthesis − and a well-known target of the SFA-induced endoplasmic reticulum stress, often linked to the hepatic pro-inflammatory microenvironment and inflammatory cells recruitment during NASH [32,36]. Macrophages are intensively involved in NASH by activating the p65−NF−κB pathway and enhancing the synthesis of reactive oxygen/nitrogen species (ROS/RNS) and inflammatory cytokines [37,38]. In the present study, it was found that the hybrid model of NASH enhances the hepatic CD68^+^ cell density and p65−NF−κB protein levels in the C57BL/6J strain while increasing the number of casp3^+^ and Ki67^+^ hepatocytes in the BALB/c strain. These findings suggest that CCl_4_ accelerates, in a strain-dependent manner, the WD-induced outcomes, and triggering distinct mechanisms of NASH progression, inducing inflammatory cell recruitment and activation of the oxidative/inflammation axis (C57BL/6J) or lipotoxicity/death and stress-related proliferation of hepatocytes (BALB/c), which are both essential for the transition of hepatic steatosis to NASH.

Through analyzing hepatic metabolomic profiles and pathways, it was found that the hybrid NASH model impairs aminoacyl-tRNA, pantothenate and CoA biosynthesis, and phenylalanine metabolism pathways, mostly related to the energetic and lipid clearance mechanisms. Similar results were obtained when assessing the hepatic metabolomic profile of C57BL/6J mice subjected to a CCl_4_-associated diet-induced NASH model [39]. Inhibition of pantothenate kinase activity reduces hepatic levels of CoA and acetyl-CoA synthesis, leading to TCAC uncoupling, lipotoxic intermediates accumulation, and electron leakage, which are well-known mechanisms of lipotoxicity, oxidative stress, and NASH progression [40]. Phenylalanine metabolism has also been proposed as a potential NASH-promoting mechanism by impairing both lipid oxidation (β-oxidation) and trafficking (lipoprotein synthesis) [41]. Collectively, the results of the enrichment analysis suggest that the combination of WD and CCl_4_ protocols might impact the lipid-redox axis dynamics in both mouse strains, leading to oxidative stress and compromising lipid clearance activity. At the metabolite level, SAH, phenylalanine, uracil, tyrosine, niacinamide, aspartate, formate, hypoxanthine, and valine were identified as potential drivers of NASH. SAH is a byproduct of the S-adenosylmethionine (SAM) metabolism, considered a donor of methyl groups in hepatic transmethylation processes [42]. In the present study, we show that the hybrid model of NASH reduces the levels of SAH, which might be linked to increased SAM levels, in agreement with other murine models of NASH [43]. The silencing activity of glycine-N-methyl transferase, which is the acting enzyme that modulates the demethylation process and leads to the synthesis of SAH, increases the SAM/SAH ratio, and triggers oxidative stress [43]. Xanthine oxidase (XO) activity has also been linked to NASH by converting hypoxanthine into uric acid and residual ROS. Pharmacological inhibition of XO reduces hepatic steatosis, lipid peroxidation of hepatocytes, and hepatic pro-inflammatory macrophage infiltration [44]. A drop in hypoxanthine levels suggests a potential underlying mechanism relying on XO-increased activity. Likewise, dietary supplementation with valine increases serum levels of triglycerides and aminotransferases, while decreasing hepatic levels of reduced glutathione and glutathione peroxidase [45]. Phenylalanine and tyrosine metabolism is pivotal for lipid trafficking dynamics, by modulating the synthesis of very low-density lipoproteins [46]. The increased levels of both aromatic amino acids could indicate the impairment of this mechanism, hence contributing to lipid accumulation in the liver. Taken together, these findings suggest that the hybrid model of NASH modulates hepatic metabolomic profiles in both mouse strains, compromising lipid synthesis/clearance and activating oxidative stress-related molecular mechanisms.

The enrichment analysis showed that the TCAC molecules and pyruvate metabolism might be related to the distinct hepatic metabolomic profile in both mouse strains. In C57BL/6J mice, hepatic levels of fumarate and uracil metabolites were downregulated, whereas levels of AMP and IMP were reduced in BALB/c mice. Fumarate is an essential metabolite that modulates TCAC activity and the mitochondrial respiratory chain. Fumarate hydratase-KO mice show fumarate accumulation, which triggers the Nrf2 pathway by inactivating Keap1 [47]. IMP has been linked to the energetic metabolism of hepatocytes, especially AMP synthesis, and the energy metabolism of mitochondria. Dietary supplementation with IMP enhances hepatic antioxidant activity by attenuating mitochondrial uncoupling activity and damage to hepatocytes [48]. Thus, we suggest that the hybrid model of NASH abruptly decreases Nrf2 protein levels in both strains by distinct underlying mechanisms. Nrf2 expression has been proposed as a potential link between hepatic oxidative stress and NASH progression, also modulating protein levels of cytoprotective enzymes, including superoxide dismutase, heme oxygenase 1, and glutathione peroxidase. Nrf2-KO mice are, indeed, more susceptible to developing NASH when submitted to a WD protocol through enhancement of protein levels of SREBP-1c, of which its activity has been related to the activation of lipid synthesis metabolism and impairment of lipid oxidation mechanisms [49,50,51].

## 5. Conclusions

The hybrid model induces a high-grade fibrosis-associated NASH in both C57BL/6J and BALB/c mice, mimicking metabolic (glucose intolerance and AT-related stress), morphologic (high-grade microvesicular steatosis and lobular inflammation and extensive fibrosis), and molecular (impaired inflammatory-redox dynamics) hallmarks of clinically relevant NASH along with strain-specific differences in metabolic profiles, suggesting a potential interplay with the p65−NF−κB/Nrf2 pathway.

## Data Availability

All of the data is contained within the article and the Supplementary Materials, authors may provide raw data upon reasonable request.

## Supplementary Materials

The following supporting information can be downloaded at: https://www.mdpi.com/article/10.3390/antiox12020290/s1, Figure S1: Experimental design; Figure S2: Assessment of chow and water/high-sugar solution (HSS) intake and body weight evolution; Figure S3: Hepatic macroscopic analysis; Figure S4: Assessment of final body weight and absolute/relative liver and adipose tissue weight; Figure S5: Determination of alanine aminotransferase (ALT) levels; Figure S6: Assessment of the glucose tolerance test (GTT) and the area under the curve (AUC); Figure S7: Identification of NASH-related parameters; Figure S8: Effects of the hybrid model of NASH on the adipose tissue; Table S1: Nutritional composition of western and basal chows.
