# Supplementary material for: Unraveling Hepatic Metabolomic Profiles and Morphological Outcomes in a Hybrid Model of NASH in Different Mouse Strains
Source: Antioxidants (Basel). 2023 Jan 27;12(2):290. doi: 10.3390/antiox12020290 (PMC9952348; doi:10.3390/antiox12020290)
Supplement: Supplementary file 1 [file antioxidants-12-00290-s001.zip › antioxidants-2050276-supplementary.pdf]

Unraveling Hepatic Metabolomic Profiles and Morphological Outcomes in a Hybrid Model of NASH in Different Mouse Strains

Gabriel P. Bacil [1], Guilherme R. Romualdo [1,2], Priscila M. F. D. Piagge [3], Daniel R. Cardoso [3], Mathieu Vinken [4], Bruno Cogliati [5] and Luís F. Barbisan [2,*]

[1] Department of Pathology, Botucatu Medical School (FMB), São Paulo State University (UNESP), Botucatu 18618-689, Brazil
[2] Department of Structural and Functional Biology, Biosciences Institute, São Paulo State University (UNESP), Botucatu 18618-689, Brazil
[3] Department of Chemistry and Molecular Physics, São Carlos Institute of Chemistry (IQSC), University of São Paulo (USP), São Carlos 13566-590, Brazil
[4] Department of Pharmaceutical and Pharmacological Sciences, University of Vrije, 1090 Brussel, Belgium
[5] Department of Pathology, School of Veterinary Medicine and Animal Science, University of São Paulo (USP), São Paulo 05508-270, Brazil

* Correspondence: luis.barbisan@unesp.br; Tel.: +55-14-3880-0469

Keywords: non-alcoholic fatty liver disease; non-alcoholic steatohepatitis; mouse strains; metabolomic profile.

Supplementary Materials (for online publication).

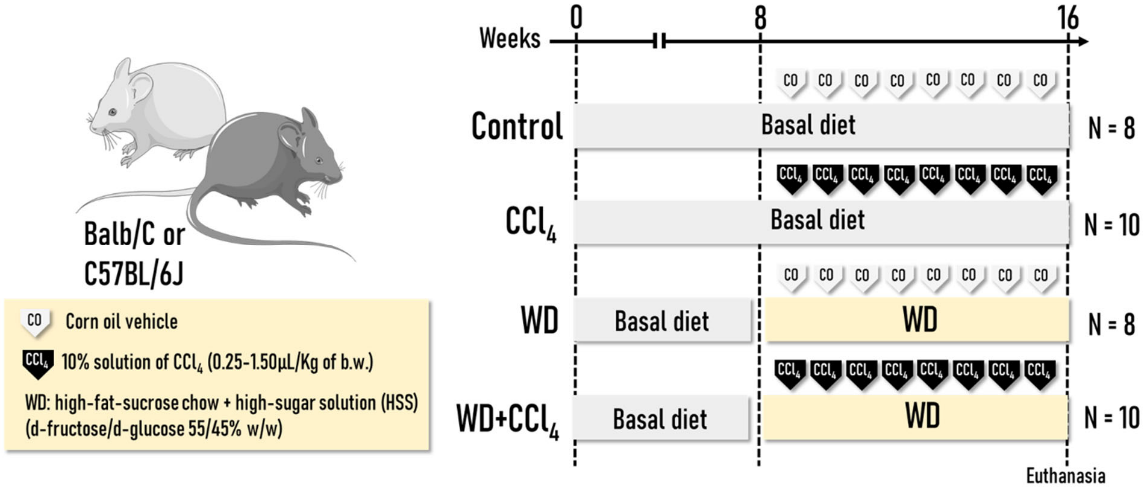

Figure S1. Experimental design of the short-period hybrid model of non-alcoholic steatohepatitis (NASH) in male C57BL/6J or BALB/c mice.

Images were acquired from the smart.servier.com.

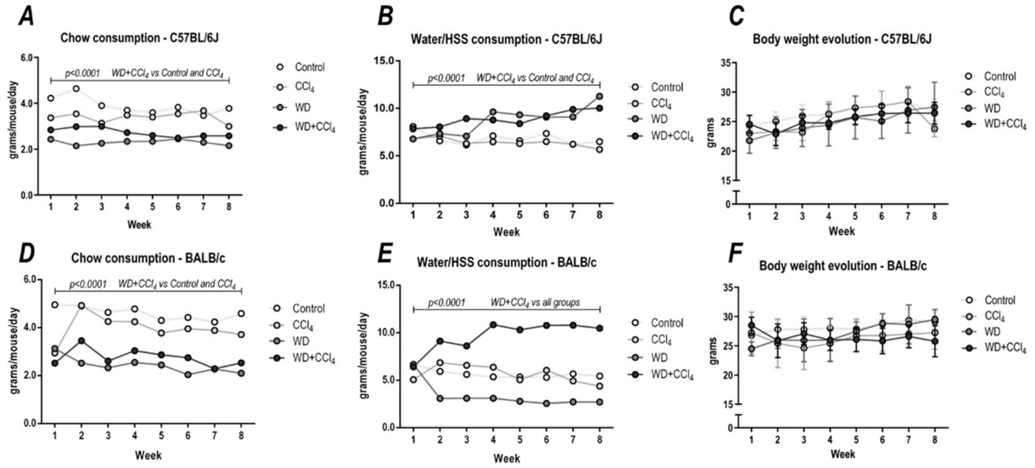

Figure S2. Effects of the hybrid model of NASH on chow, water, and high-sugar consumption (HSS) and body weight evolution. **(A and D)** Basal and high-fat chow consumption of C57BL/6J and BALB/c mice. **(B and E)** Water and HSS consumption of C57BL/6J and BALB/c mice. **(C and F)** Body weight evolution of C57BL/6J and BALB/c mice. Data were analyzed by one-way ANOVA and Tukey *post hoc* test. Data were presented as mean or mean ± standard deviation (S.D.) values, according to the time point, and were considered significantly different when $p<0.05$. Control and WD: 8 animals/group; CCl_4, and WD+CCl_4: 10 animals/group. WD: a high-fat/sucrose chow (20% of fat/sucrose) and high-sugar solution (HSS, D-fructose/D-glucose or 23.1 and 18.9g/L) for drinking. CCl_4: intraperitoneal (i.p.) injections of 10% diluted oil solution of CCl_4 (0.25-1.50μL/g of body weight, 3×/week).

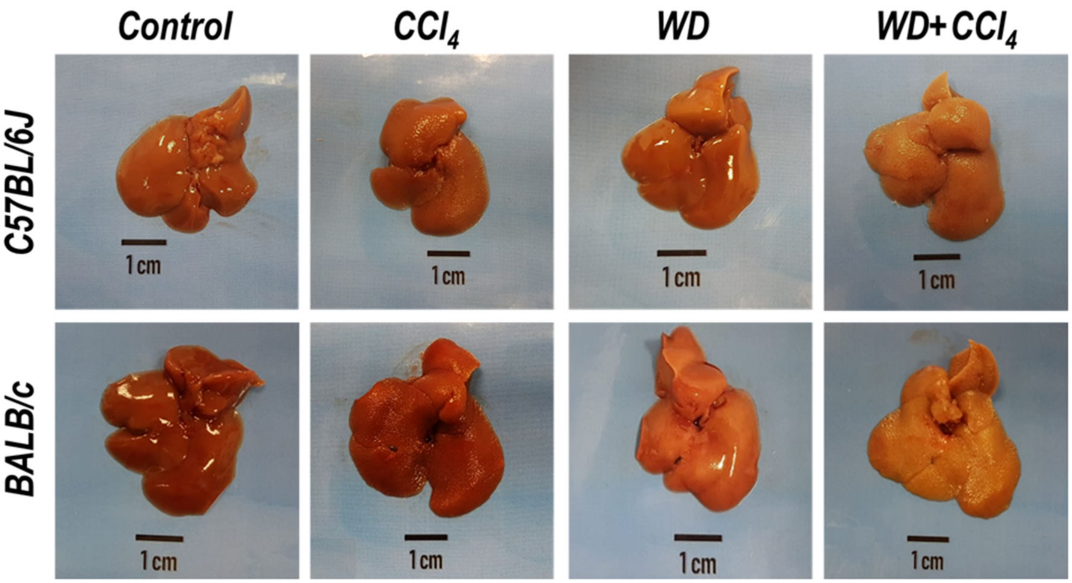

Figure S3. Representative macroscopic overview of the liver of C57BL/6J and BALB/c mice subjected to the hybrid model of NASH. WD: a high-fat/sucrose chow (20% of fat/sucrose) and high-sugar solution (HSS, d-fructose/d-glucose or 23.1 and 18.9g/L) for drinking. CCl_4: intraperitoneal (i.p.) injections of 10% diluted oil solution of CCl_4 (0.25-1.50μL/g of body weight, 3×/week).

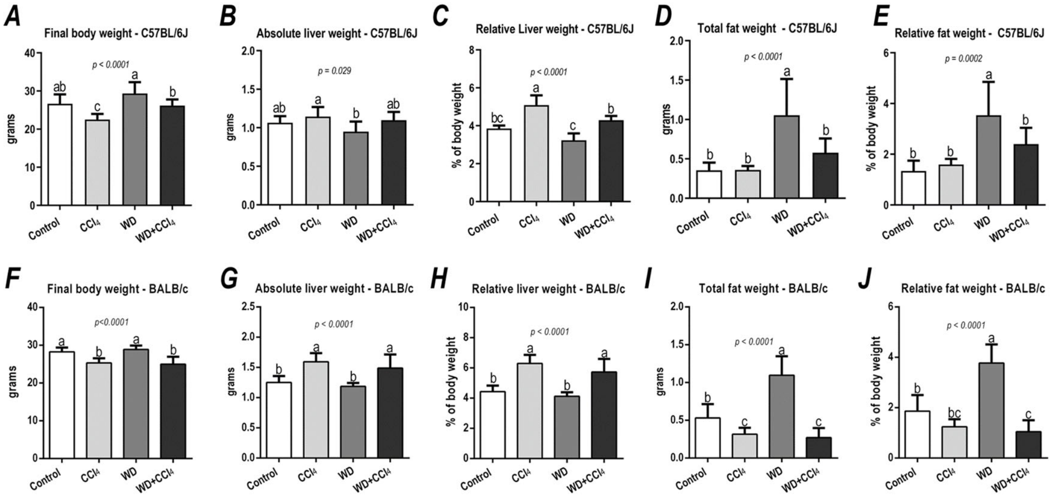

Figure S4. General findings of C57BL/6J and BALB/c mice subjected to the hybrid model of NASH. **(A and F)** Final body weight of C57BL/6J and BALB/c. **(B and G)** Absolute liver weight of C57BL/6J and BALB/c. **(C and H)** Relative liver weight of C57BL/6J and BALB/c. **(D and I)** Total fat weight of C57BL/6J and BALB/c. **(E and J)** Relative fat weight (total) of C57BL/6J and BALB/c. Data were analyzed by one-way ANOVA and Tukey *post hoc* test. Data were presented as mean ± standard deviation (S.D.). Different letters correspond to significant differences ($p<0.05$) among groups. Control and WD: 8 animals/group; and CCl_4 and WD+CCl_4: 10 animals/group. WD: a high-fat/sucrose chow (20% of fat/ sucrose) and high-sugar solution (HSS, D-fructose/D-glucose or 23.1 and 18.9g/L) for drinking. CCl_4: intraperitoneal (i.p.) injections of 10% diluted oil solution of CCl_4 (0.25-1.50μL/g of body weight, 3×/week).

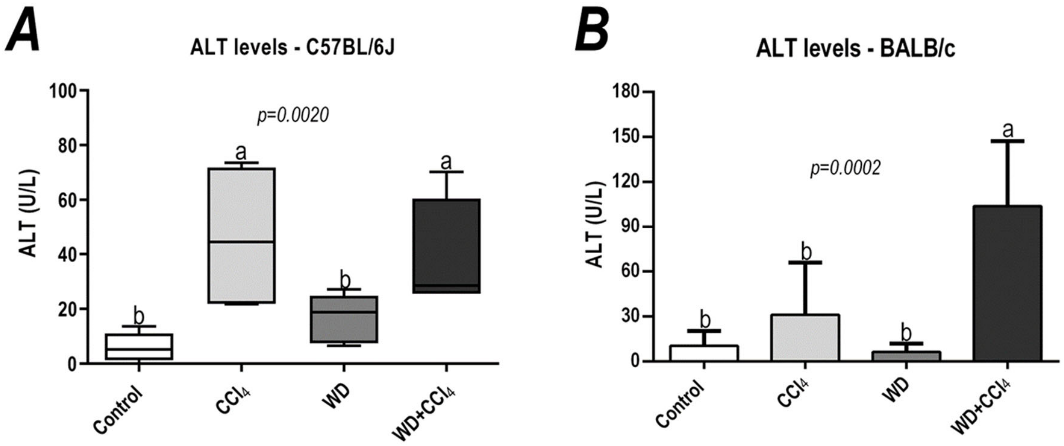

Figure S5. Determination of alanine aminotransferase (ALT) levels in serum samples from **(A)** C57BL/6J and **(B)** BALB/c mice subjected to the hybrid model of NASH, respectively. Data were analyzed by one-way ANOVA or Kruskal-Wallis and Tukey *post hoc* test and presented as mean ± standard deviation (S.D.) or median ± (max. and min.). Different letters correspond to significant differences ($p<0.05$) among groups. Control, CCl_4, WD, and WD+CCl_4: 5 animals/group. WD: a high-fat/sucrose chow (20% of fat/sucrose) and high-sugar solution (HSS, D-fructose/D-glucose or 23.1 and 18.9g/L) for drinking. CCl_4: intraperitoneal (i.p.) injections of 10% diluted oil solution of CCl_4 (0.25-1.50µL/g of body weight, 3×/week).

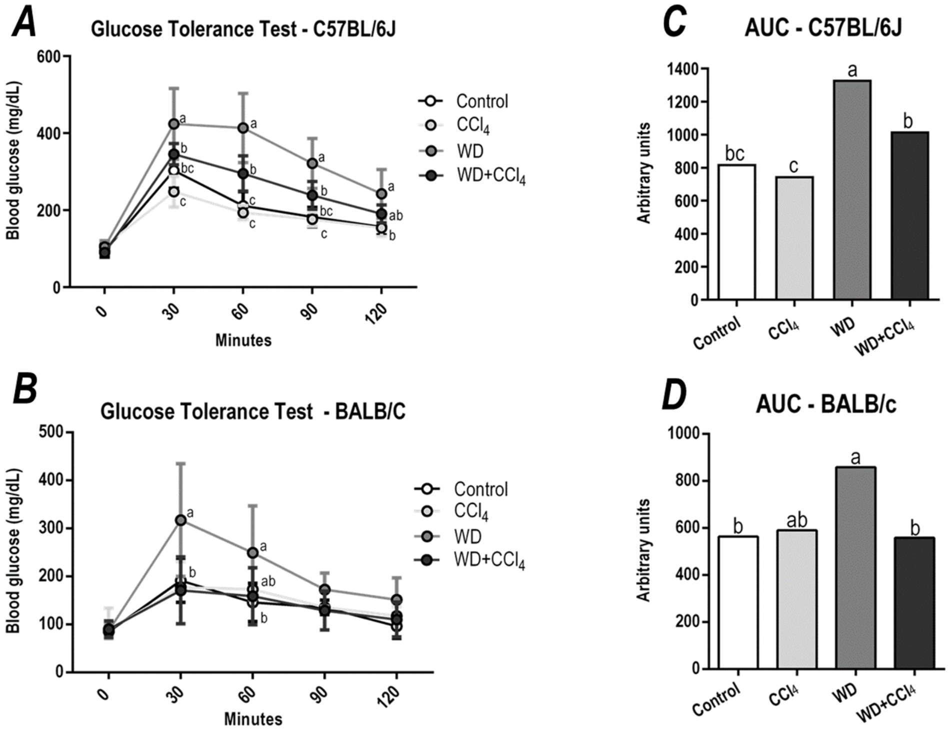

Figure S6. (A and B) Glucose tolerance test (GTT) and **(C and D)** area under the curve (AUC) of C57BL/6J and BALB/c mice subjected to the hybrid model of NASH. Data were analyzed by two-way ANOVA and Tukey *post hoc* test. Data were presented as mean ± standard deviation (S.D.), according to the time point. Different letters correspond to significant differences ($p<0.05$) among groups. Control, WD, CCl_4, and WD+CCl_4: 6 animals/group. WD: a high-fat/sucrose chow (20% of fat/sucrose) and high-sugar solution (HSS, D-fructose/D-glucose or 23.1 and 18.9g/L) for drinking. CCl_4: intraperitoneal (i.p.) injections of 10% diluted oil solution of CCl_4 (0.25-1.50μL/g of body weight, 3×/week).

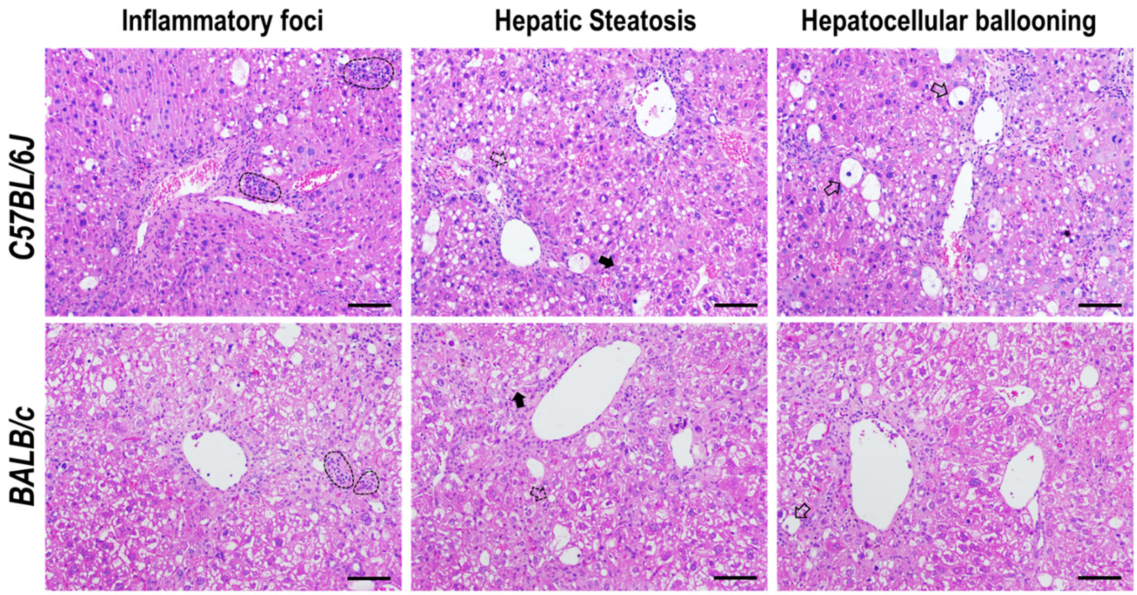

Figure S7. Representative photomicrograph of hematoxylin & eosin (H&E)-stained hepatic sections (200× objective, scale bar=50μm) of C57BL/6J and BALB/c mice subjected to the hybrid model of NASH (WD+CCl_4 groups). Identification of NASH-related morphological features (inflammatory foci occurrence, hepatic steatosis, and hepatocellular ballooning). Dotted outlines indicate inflammatory foci, filled arrows indicate microvesicular steatosis, dotted arrow indicates macrovesicular steatosis, and unfilled arrows indicate hepatocellular ballooning.

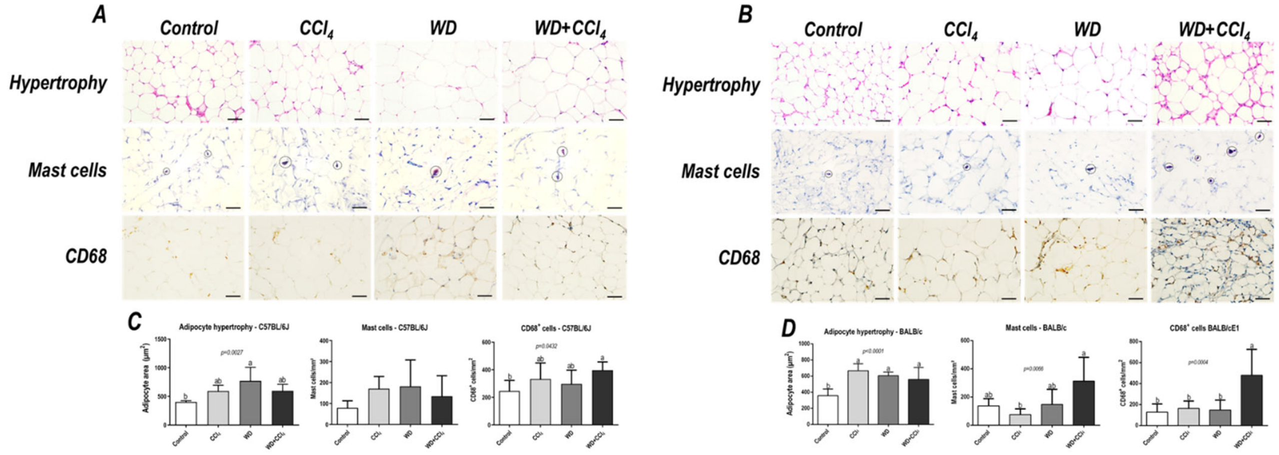

Figure S8. Representative photomicrograph of adipose tissue (AT) sections immunoreacted for CD68 and stained with hematoxylin & eosin (HE) and toluidine blue (200× objective, scale bar=50μm) of **(A)** C57BL/6J and **(B)** BALB/c subjected to the hybrid model of NASH. **(C and D)** Assessment of adipocyte hypertrophy, mast cells (MC) density, and CD68+ cells/mm_2 of C57BL/6J and BALB/c mice. Dotted outlines indicate the mast cells. Data were analyzed by one-way ANOVA and Tukey *post hoc* test. Data were presented as mean ± standard deviation (S.D.). Different letters correspond to significant differences ($p<0.05$) among groups. Control and WD: 6 animals/group; and CCl_4 and WD+CCl_4: 9 animals/group. WD: a high-fat/sucrose chow (20% of fat /sucrose) and high-sugar solution (HSS, D-fructose/D-glucose or 23.1 and 18.9g/L) for drinking. CCl_4: intraperitoneal (i.p.) injections of 10% diluted oil solution of CCl_4 (0.25-1.50μL/g of body weight, 3×/week).

Table S1. Nutritional composition of basal and high-fat/sucrose chow.

	Dietary nutrients	Basal chow	High-fat/sucrose chow
	Protein	220	235
Carbohydrates	Corn starch	554	202
	Sucrose	-	202
Fatty acids	Soy oil	40	-
	Corn oil	-	50
	Lard	-	200
Fibers, vitamins, and minerals	Fibers	70	50
	Mineral mix	90	90
	Vitamin mix	10	10
	**Total (g)**	**1000**	**1000**
	**Total (kcal/g)**	**3456**	**4806**
